# Esophageal function and non-acid reflux evaluated by impedance-24 h-pH-metry, high-resolution manometry, and gastroscopy after one-anastomosis gastric bypass—outcomes of a prospective mid-term study

**DOI:** 10.1007/s00464-022-09857-9

**Published:** 2023-01-24

**Authors:** D. M. Felsenreich, M. L. Zach, N. Vock, J. Jedamzik, J. Eichelter, M. Mairinger, L. Gensthaler, L. Nixdorf, P. Richwien, C. Bichler, I. Kristo, F. B. Langer, G. Prager

**Affiliations:** Division of Visceral Surgery, Department of General Surgery, Medical University of Vienna, Waehringer Guertel 18-20, Vienna, 1090 Austria

**Keywords:** One-Anastomosis Gastric Bypass, Impedance-24 h-pH-metry, High-resolution manometry, Gastroscopy, Non-acid reflux, Quality of life

## Abstract

**Background:**

One-Anastomosis Gastric Bypass (OAGB) is the third most common bariatric operation for patients with obesity worldwide. One concern about OAGB is the presence of acid and non-acid reflux in a mid- and long-term follow-up. The aim of this study was to objectively evaluate reflux and esophagus motility by comparing preoperative and postoperative mid-term outcomes.

**Setting:**

Cross-sectional study; University-hospital based.

**Methods:**

This study includes primary OAGB patients (preoperative gastroscopy, high-resolution manometry (HRM), and impedance-24 h-pH-metry) operated at Medical University of Vienna before 31st December 2017. After a mean follow-up of 5.1 ± 2.3 years, these examinations were repeated. In addition, history of weight, remission of associated medical problems (AMP), and quality of life (QOL) were evaluated.

**Results:**

A total of 21 patients were included in this study and went through all examinations. Preoperative weight was 124.4 ± 17.3 kg with a BMI of 44.7 ± 5.6 kg/m^2^, total weight loss after 5.1 ± 2.3 years was 34.4 ± 8.3%. In addition, remission of AMP and QOL outcomes were very satisfactory in this study. In gastroscopy, anastomositis, esophagitis, Barrett´s esophagus, and bile in the pouch were found in: 38.1%, 28.3%, 9.5%, and 42.9%. Results of HRM of the lower esophageal sphincter pressure were 28.0 ± 15.6 mmHg, which are unchanged compared to preoperative values. Nevertheless, in the impedance-24 h-pH-metry, acid exposure time and DeMeester score decreased significantly to 1.2 ± 1.2% (*p* = 0.004) and 7.5 ± 8.9 (*p* = 0.017). Further, the total number of refluxes were equal to preoperative; however, the decreased acid refluxes were replaced by non-acid refluxes.

**Conclusion:**

This study has shown decreased rates of acid reflux and increased non-acid reflux after a mid-term outcome of primary OAGB patients. Gastroscopy showed signs of chronic irritation of the gastrojejunostomy, pouch, and distal esophagus, even in asymptomatic patients. Follow-up gastroscopies in OAGB patients after 5 years may be considered.

**Graphical Abstract:**

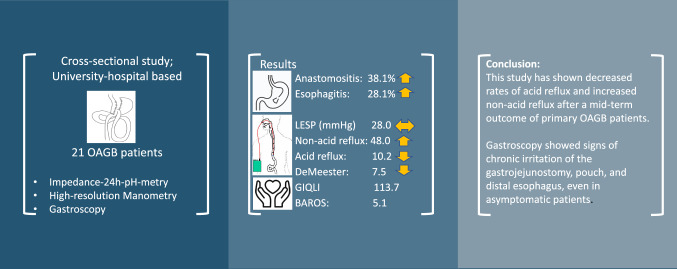

Obesity has continuously increased worldwide over the last decades and so has its associated medical problems (AMP) [1]. Especially AMP decrease patients’ life expectancy and quality of life (QOL) [2, 3]. At present, bariatric/metabolic surgery is the best treatment for both, weight loss and remission of AMP [4, 5]. Besides Sleeve Gastrectomy (SG) and Roux-en-Y Gastric Bypass (RYGB), One-Anastomosis Gastric Bypass (OAGB) is a valid and approved procedure (approved by the International Federation for the Surgery of Obesity and Metabolic Disorders (IFSO)) [6, 7].

For OAGB, a long and narrow pouch and a gastrojejunostomy with a ~ 150 cm biliopancreatic limb (BPL) are created. Further, the BPL is attached to the distal 6 cm of the pouch to prevent biliary reflux to the pouch (anti-reflux sutures) [8]. Besides the advantages of OAGB such as short operation time and good and accurate weight loss [9], an ongoing discussion focuses on the presence and severity of non-acid reflux [10]. Most studies in the literature focus on rates of revisional surgery to RYGB, gastroscopy, or questionnaire results to evaluate biliary reflux after OAGB; however, these studies only report selective measurements and snapshots [11, 12]. Impedance-24 h-pH-metry may extend the diagnosis as non-acid reflux in the distal esophagus can be measured over an entire day [13]. Currently, the present study is the largest series with the longest follow-up comparing pre- and postoperative results of esophageal functional testing in OAGB patients.

The aim of this study was to evaluate acid and non-acid reflux and esophagus motility, comparing high-resolution manometry (HRM), impedance-24 h-pH-metry and gastroscopy before and after OAGB in a mid-term follow-up. Additionally, weight loss, remission of AMPs, and QOL after OAGB were evaluated.

## Patients and methods

In total, 619 patients had primary OAGB at Medical University of Vienna before December 31, 2017. Out of these 619 patients, 112 had complete results of preoperative gastroscopy, impedance-24 h-pH-metry, and HRM preoperative. A total of 21 patients agreed to participate in this study to perform these examinations again. Additionally, data on patients’ history of weight, remission of AMP, as well as gastro-esophageal reflux disease (GERD) and QOL were gathered.

Patients with previous bariatric operations or reoperations after the OAGB were not included in this study as this could influence the outcome. Also, none of the included patients suffered from GERD prior to surgery, as this represents a contraindication to performing OAGB at our bariatric center. Hiatal hernias in the preoperative gastroscopy were explored intraoperatively, and a hiatoplasty was performed if indicated.

For this study, a minimal follow-up of three years was decided to prevent depicting any short-term effects. Mean follow-up was 5.1 ± 2.3 years. The follow-up rates for gastroscopy, impedance-24 h-pH-metry, and HRM were 100% (21/21 patients) and for the QOL questionnaires 81% (17/21 patients).

The review board of the local ethical committee of Medical University of Vienna approved this study with the reference number: 1752/2019. An Informed consent was obtained from all participating patients.

## Surgical technique

The surgical technique of OAGB was standardized in all patients and previously described in detail [8]. Thus, only some brief facts are presented here:

After the placement of the trocars, the lesser sac is opened at the smaller curvature of the stomach about 1–2 cm below the incisura angularis. Then, the stomach is partially transected horizontally with a 45 mm stapler. Next, the left crus of the diaphragm is dissected free. In patients with a preoperatively detected hiatal hernia in the gastroscopy, both crura are visualized. In patients with a verified hiatal hernia, a posterior hiatoplasty (and anterior, if necessary) is performed after complete mobilization and dissection of the distal esophagus.

Next, a 12 mm (36 French) bougie is introduced and a long and narrow pouch created using four 60 mm stapler cartridges. The angle of Treitz is identified and 150 cm (up to 250 cm in this collective) BPL is measured to create a linear end-to-side gastrojejunostomy using a 30 mm cartridge. Next, a non-absorbable running “anti-reflux” suture is placed between the end of the BPL and the distal 6 cm of the vertical staple line of the pouch. Petersen’s space is closed and the alimentary limb is attached to the antrum of the remnant stomach with a non-absorbable single-knot suture to prevent kinking of the alimentary limb (thus, avoiding pooling of bile in the pouch leading to biliary reflux). Finally, an air-leakage test of the gastrojejunostomy is performed with continuous air insufflation by gastroscopy.

## History of weight and associated medical problems

Weight and BMI at the time of the surgery, at nadir, and at the time of the follow-up were examined. Change of the BMI, excess weight loss (EWL) in %, as well at the total weight loss (TWL) in % were calculated.

The following AMP were evaluated at OAGB and at the time of the follow-up based on the need of medication: type 2 diabetes mellitus (T2D), arterial hypertension, obstructive sleep apnea syndrome (OSA), and hyperlipidemia. For this study, patients were interviewed about GERD symptoms, and their need of daily proton-pump inhibitors was noted.

## Gastroscopy

All patients received a gastroscopy before the OAGB, where standardized biopsies from the antrum, corpus, and the gastro-esophageal junction (according to the Seattle protocol [14]) were taken. All saliences as well as hiatal hernias were recorded. Helicobacter pylori infections were eradicated preoperatively.

Postoperative gastroscopies were performed specifically for this study and, again, biopsies from the anastomosis, the pouch, and the gastro-esophageal junction were performed. A special focus was set on the detection of anastomotic ulcers and bile in the pouch; however, data on other pathological issues were collected as well.

## High-resolution manometry

Patients were assessed in HRM by doing ten liquid swallow bolus trials. The pressure of the lower esophageal sphincter (LESP) and the time a liquid bolus needs for transit were measured. Further, the integrated relaxation pressure (IRP) and the distal contractile integral (DCI) were measured. IRP and DCI represent the lowest pressure at the gastrointestinal junction during opening and the power of the peristaltic waves of the esophagus during bolus transit [15]. All reference levels are listed in Table 4.

## Impedance-24 h-pH-metry

Immediately after HRM, the impedance-24 h-pH-metry probe was placed and left for 24 h. First, the acid exposure time is measured, which implies the percentage of time in 24 h that the pH value in the distal esophagus is below 4. Further, the total number of refluxes in 24 h from the stomach/pouch to the esophagus can be evaluated and also divided into acid and non-acid refluxes. Non-acid refluxes can indicate biliary reflux. Also, the DeMeester score is calculated based on duration and number of acid refluxes in different body positions [16]. Again, all reference levels are listed in Table 4.

## Questionnaires

Patients in this study were asked to complete four questionnaires: Short Form 36 (SF-36), Gastrointestinal Quality of Life Index (GIQLI), Bariatric Quality of Life index (BQL), and Bariatric Analysis and Reporting Outcome System (BAROS). Preoperative questionnaire results were not available.

SF-36 is a validated questionnaire examining patients’ general QOL in 36 items. In eight sections about physical and psychological health, a score between 0 and 100 can be reached [17]. GIQLI was developed by Eypasch E. et al. in 1993 consisting of 36 questions with a total score of 144 points. This questionnaire focusses on all kinds of patients’ gastrointestinal symptoms including GERD, flatulence, abdominal pain, bowel movement, diarrhea, etc. [18]. Further, BQL was developed in 2005 and updated in 2009 by Weiner S. et al. and is a practical tool to evaluate QOL, especially in patients undergoing bariatric and metabolic surgery. The questionnaire consists of 13 items, with a total of 65 points equaling 100% [19]. Finally, BAROS is an outcome score to evaluate the success of a bariatric/metabolic procedure. Beside weight loss, QOL, remission of comorbidities, also complications, and reoperations are considered. Using the scoring key, the outcome can be classified as failure, fair, good, very good, or excellent. As all patients had at least one AMP, only “BAROS with Comorbidity” was used [20].

## Statistical analysis

Due to the descriptive nature of this study, a power analysis was not necessary, and additionally, all patients suitable to the inclusion criteria were considered for participation in this study.

The descriptive data in this study were presented as percentage or mean with standard deviation. The comparisons of the functional testing before and after surgery were performed using students t test with a *p* value of < 0.05 considered as significant. SPSS® v27 for Windows® (IBM Corporation, Armonk, New York, USA) was used for statistical calculations.

## Results

This study includes all patients with gastroscopy, HRM, and impendance-24 h-pH-metry before and after primary OAGB. A total of 21 patients were willing to take part in these examinations. None of them had a previous operation before the OAGB. Two patients received concomitant hiatal hernia repair due to preoperatively detected and intraoperatively verified hiatal hernias. Further baseline characteristics are presented in Table 1.

**Table 1:** Baseline characteristics and history of weight

	All patients
	(*n* = 21)
Sex (female) (*n = *15)	71.4%
Age at OAGB (years)	43.1 ± 10.3
Previous bariatric procedures	0%
OAGB	
Length biliopancreatic limb (cm)	150–250
Hiatoplasty (n = 2)	9.5%
Anti-reflux sutures (n = 21)	100%
Weight at OAGB:	
Weight (kg)	124.4 ± 17.3
BMI (kg/m^2^)	44.7 ± 5.6
Nadir weight:	
Weight (kg)	75.8 ± 10.8
BMI (kg/m^2^)	27.3 ± 2.9
Change BMI	17.4 ± 3.8
TWL (%)	39.0 ± 7.9
EWL (%)	95.3 ± 15.7
Follow-up	
Minimal follow-up (years)	3.0
Mean Follow-up (years)	5.1 ± 2.3
Weight at follow-up	
Weight (kg)	81.6 ± 15.2
BMI (kg/m^2^)	29.3 ± 5.2
Change BMI	15.4 ± 4.2
TWL (%)	34.4 ± 8.3
EWL (%)	78.7 ± 20.9

*OAGB* one-anastomosis gastric bypass; *BMI* body mass index

### Weight loss and remission of associated medical problems

Mean weight and BMI at the time of the OAGB were 124.4 ± 17.3 kg and 44.7 ± 5.6 kg/m^2^. The lowest postoperative weight and BMI these patients were able to reach were 75.8 ± 10.8 kg and 27.3 ± 2.9 kg/m^2^, which is equivalent to a TWL of 39.0 ± 7.9%. Mean weight and BMI after the end of the follow-up period of 5.1 ± 2.3 years were 81.6 ± 15.2 kg and 29.3 ± 5.2, with a TWL of 34.4 ± 8.3%. Further weight loss results can be found in Table 1. In this small series, no difference between patients with a BPL of 150 cm and 250 cm was found; however, only two patients had a BPL of 250 cm.

Remission of AMP within the follow-up period was observed as follows: Remission of T2D was noted in five out of six patients (83.3%), and of arterial hypertension in four out of ten patients (40.0%). Additionally, all three out of three patients (100%) with OSA and seven out of seven patients (100%) with hyperlipidaemia experienced remission of the disease. None of these patients were suffering from GERD at the time of the OAGB; nevertheless, at the end of the follow-up period, three out of 21 (14.3%) are suffering from GERD and are successfully treated with proton-pump inhibitors for symptom control (Table 2).

**Table 2:** Associated medical problems and GERD before OAGB and at follow-up

	All patients
Basis (OAGB)	(*n* = 21)
T2D	6 (28.6%)
Arterial hypertension	10 (47.6%)
OSA	3 (14.3%)
Hyperlipidemia	7 (33.3%)
GERD	0 (0%)
Follow-up	(*n* = 21)
T2D	1 (4.7%)
Arterial hypertension	6 (28.6%)
OSA	0 (0%)
Hyperlipidemia	0 (0%)
GERD	3 (14.3%)

*OAGB* one-anastomosis gastric bypass; *T2D* diabetes mellitus type 2; *OSA* obstructive sleep apnoea; *GERD* gastro-esophageal reflux disease

### Gastroscopy

In the preoperative gastroscopy, five (19%) patients had antrum gastritis and two (9.5%) patients’ mild esophagitis. In six patients, a small hiatal hernia with a mean size of 1.67 cm (Range: 1–3 cm) was detected; nevertheless, after intraoperative visualization of both crura, only two of them were verified intraoperatively and treated with hiatal hernia repair. All four patients with helicobacter pylori had an eradication treatment before the operation.

In the gastroscopy for this study, at the end of the follow-up period, pouchitis in six patients (28.6%) and bile in the pouch in nine patients (42.9%) were detected. However, none of these nine patients had bile in the distal esophagus. The anastomositis rate was 23.8% (five patients) macroscopic and 38.1% (eight patients) microscopic (biopsies); three of these patients also had asymptomatic anastomotic ulcers. The esophagitis rate was 14.3% (three patients) macroscopic and 28.3% (six patients) microscopic (biopsies). Interestingly, two patients (9.5%) with Barrett´s esophagus were detected, who did not have it preoperatively. Further results of the macroscopic and microscopic findings of the preoperative and postoperative gastroscopies are presented in Table 3.

**Table 3:** Gastroscopy before OAGB and at follow-up

	Alle patients
*Basis (OAGB)*	(*n* = 21)
Macroscopic	
Gastritis	5 (19.0%)
Esophagitis	2 (9.5%)
Hiatal hernia	6 (28.6%)*
Mean hiatal hernia size	1.67 cm (R 1–3)
Microscopic	4 (19.0%)**
Helicobacter pylori	4 (19.0%)
Gastritis	1 (4.8%)
Esophagitis	0 (0%)
Barrett´s esophagus	
*Follow-up*	(*n* = 21)
Macroscopic	
Pouchitis	6 (28.6%)
Anastomositis	5 (23.8%)
Anastomotic ulcer	3 (14.3%)
Esophagitis	3 (14.3%)
Hiatal hernia	0 (0.0%)***
Bile in the pouch	9 (42.9%)
Microscopic	
Helicobacter pylori	0 (0%)
Anastomositis	8 (38.1%)
Esophagitis	6 (28.3%)
Barrett`s esophagus	2 (9.5%)

*OAGB* one-anastomosis gastric bypass; *R* range

*All patients with hiatal hernia in the preoperative gastroscopy were explored intraoperatively and a hiatoplasty was performed if indicated

**Helicobacter pylori infections were eradicated preoperatively and verified by stool test

***Hiatal hernias are hard to detect in patients with a proper pouch where inversion with the gastroscope is not possible

### High-resolution manometry and impedance-24 h-pH-metry

Comparing data gained from HRM before and 5.1 years after primary OAGB, one can see that LESP did not change significantly: 25.5 ± 10.7vs. 28.0 ± 15.6 mmHg; *p* = 0.576. The time of a swallowed liquid bolus transferring through the esophagus decreased significantly (7.2 ± 1.8 vs. 4.7 ± 2.2 s; *p* = 0.001); nevertheless, both values are within the normal range.

Comparing the results of the impedance-24 h-pH-metry before and 5.1 years after primary OAGB, acid exposure time of the esophagus decreases significantly from 4.1 ± 3.9 to only 1.2 ± 1.2%; *p* = 0.004. Further, the total number of refluxes in 24 h was stable with 52.1 ± 20.8 (preoperatively) and 58.2 ± 32.1 (at follow-up), but the composition had changed. While the number of non-acid refluxes doubled (24.0 ± 15.2 to 48.0 ± 29.4; *p* = 0.003), the number of acid refluxes more than halved (28.1 ± 19.4 to 10.2 ± 8.7; *p* = 0.001). The DeMeester score, which mainly reflects acid reflux, also decreased significantly from 17.5 ± 15.7 to a normal level of 7.5 ± 8.9; *p* = 0.017. Further results of functional esophageal testing can be seen in Table 4.

**Table 4:** Functional testing (HRM and impedance-24 h-pH-metry) before OAGB and at follow-up

All patients			
	Basis OAGB (*n* = 21)	Follow-up (*n* = 21)	*p*-value
Manometry			
LESP (mmHg) (10-35 mmHg)	25.5 ± 10.7	28.0 ± 15.6	0.576
Time liquid bolus (s) (< 12 s)	7.2 ± 1.8	4.7 ± 2.2	**0.001**
IRP (mmHg) (< 15 mmHg)	13.6 ± 4.5	11.5 ± 5.8	0.244
DCI (mmHg-cm-s) (450—8000 mmHg-cm-s)	2546.6 ± 1929.5	1410.7 ± 923.9	**0.036**
Impedance-24 h-pH-metry			
Acid exposure time (% of 24 h) (normal < 4.2%)	4.1 ± 3.9	1.2 ± 1.2	**0.004**
Total number of refluxes (normal < 40)	52.1 ± 20.8	58.2 ± 32.1	0.479
Number non-acid refluxes	24.0 ± 15.2	48.0 ± 29.4	**0.003**
Number acid refluxes	28.1 ± 19.4	10.2 ± 8.7	**0.001**
DeMeester score (normal 14.72)	17.5 ± 15.7	7.5 ± 8.9	**0.017**

*OAGB* one-anastomosis gastric bypass; *HRM* high-resolution manometry; *LESP* lower esophageal sphincter pressure; *IRP* integrated relaxation pressure; *DCI* distal contractile integral; *s* seconds

### Quality of life

The results in the eight categories of the SF-36 questionnaire are displayed in Table 5. Further, in BQL and GIQLI, patients were able to reach 54.2 ± 7.9 and 113.7 ± 14.0 points. The score of BAROS was 5.1 ± 1.6, which equals a “very good” outcome in the corresponding key.

**Table 5:** Quality of life and bariatric outcome before OAGB and at follow-up

All patients	
Follow-up	(*n* = 17)
*SF-36*	
PF (Physical Functioning)	83.2 ± 19.8
RP (Role Physical)	89.7 ± 21.8
BP (Bodily Pain)	73.8 ± 29.5
GH (General Health)	78.6 ± 17.3
VT (Vitality)	65.4 ± 23.7
SF (Social Functioning)	86.8 ± 19.0
RE (Role Emotional)	94.1 ± 17.6
MH (Mental Health)	81.4 ± 11.8
*BQL*	54.2 ± 7.9
*GIQLI*	113.7 ± 14.0
*BAROS* (with comorbidities)	5.1 ±1.6

*SF-36* short form-36; *BQL* bariatric quality-of-life index; *GIQLI* gastrointestinal quality-of-life index; *BAROS* bariatric analysis and reporting outcome system

## Discussion

This mid-term outcome study presents results of gastroscopy, HRM, and impedance-24-pH-metry before and after primary OAGB. Additional data on weight loss, remission of AMP, and QOL were gathered. The main findings of this study were high rates of esophagitis, anastomositis, bile in the pouch, and also Barrett’s esophagus in two patients. While acid exposure of the distal esophagus had decreased after primary OAGB, the rate of non-acid reflux had increased.

The 21 patients included in the present study are a subgroup of all patients that received OAGB at the Medical University of Vienna. In 2021, Jedamzik J. et al. published data on conversion rates and reasons for the conversions as well as data on weight loss of all OAGB patients operated at the Medical University of Vienna before 2019. The overall conversion rate was 7.7%, whereas the reasons for the conversion were reflux in 4.1%, marginal ulcers in 1.1%, stenosis in 1.0%, malnutrition in 0.9%, and weight regain in 0.3% [12].

### Weight loss and remission of associated medical problems

In terms of postoperative weight loss and remission of AMP after a mean follow-up of 5.1 years, the results of this study may be rated a great success. Nevertheless, when focusing on the nadir weight, the initial EWL was about 95.3%, which means that a small number of patients in this study had an initial EWL of more than 100%. To clarify, none of the patients in this study were suffering from malnutrition at any point. In fact, a BPL of more than 200 cm (standard BPL 150 cm) has been considered too long for some time; however, a few of the patients in this study still had a OAGB with a BPL of > 200 cm as common at the time. In the literature, comparable results in terms of weight loss and remission of AMP can be found, e.g., in a study by Hussain A. et al. including 519 primary OAGB: the mean EWL was 89% after the first year and 77% after three years. Remission rates of AMP between 55 and 99% after three years were observed [21].

### Acid/biliary reflux

Despite the fact that biliary reflux after OAGB is one of the hot topics discussed at every expert meeting, studies dealing with this issue are rare. Most studies describing biliary reflux after OAGB are based on patients with the need of conversion to RYGB (adding a jejuno-jejunostomy). These studies report conversion rates between 1.2 and 4.1% due to biliary reflux [12, 22]. However, in some of these studies, objective preoperative diagnostics proving biliary reflux were missing. Experiences from our bariatric center in terms of symptomatic biliary reflux after OAGB showed that in most patients with the need of a conversion to RYGB, a blockage of the drainage in the common limb was observed, e.g., kinking of the small bowel, adhesions, and Petersen´s space hernia. [12].

Another examination used to diagnose biliary reflux after OAGB was gastroscopy to detect any signs for irritation of the pouch and esophagus (pouchitis, esophagitis, Barrett´s esophagus, etc.) or bile in the pouch/esophagus. The weakness of this examination in this regard is that the lesions in the pouch/esophagus can be caused by any composition of the reflux (acid or biliary). In addition, bile detected in the pouch is just a snapshot and cannot provide any information on the frequency of the biliary reflux and the circumstances when it occurred (e.g., standing up, lying down, after food intake, during the night, etc.).

Saarinen T. et al. performed gastroscopies and scintigraphy six months after OAGB in a prospective study and reported abnormal findings such as esophagitis, stromal ulcers, and inflammation of the pouch in 39.5% of their patients. Bile in the pouch was not reported in the gastroscopy, but results of the scintigraphy reported biliary reflux up to the pouch in 31.6% of the patients [11]. These findings are in line with the results of the present study where irritation of the anastomosis, pouch, and GE junction as well as bile in the pouch in over 40% were detected. Performing a scintigraphy for the diagnosis of biliary reflux after OAGB has clear advantages over gastroscopy as the tracer is specific for bile and the duration of the examination is between 1 and 2 h to detect biliary reflux episodes [11]. Nevertheless, information about reflux occurring during the night and in different body positions cannot be detected.

The probably most potent objective measurement at detecting biliary reflux available today is impedance-24 h-pH-metry, especially if combined with HRM for additional information about the swallowing act and esophagus motility. This examination provides data about acid and non-acid refluxes during 24 h. Not only are the total number of refluxes in 24 h recorded but also the length of the episodes and the different situations in which refluxes occur (in upright position, lying down, after food intake, during sleep, etc.). These data are especially interesting for individual patients with reflux problems.

The results of this study support the assumption that primary OAGB is a procedure that decreases acid reflux significantly but, on the other hand, increases non-acid reflux, which is a relatively strong indicator for biliary reflux. The results of the impedance-24 h-pH-metry also show that bile has a potential to flow all the way up from the small bowel to the esophagus, passing the gastrojejunostomy, pouch, and the lower esophageal sphincter (LES) as the measurement is performed at the level of the distal esophagus immediately above the ora serrata. The fact that the LESP (as well as all other values of the HRM) was in the normal range and only slightly changed compared to preoperative data, indicates that the LES was not harmed during the OAGB procedure.

In the literature, there is a study by Nemeth W.H. et al. that also used 24 h-pH-metry but at 64 months after OAGB in 43 patients. The authors divided participants into those with acid, biliary, and mixed reflux; 30.2% of their patients were suffering from acid reflux and 27.9% from biliary reflux, while biliary reflux activity was equal compared to our study, acid activity, and DeMeester score were much higher. First, this may have been caused by the fact that the authors also included 34.8% revisional OAGB patients (most of whom were converted from adjustable gastric banding), and, second, by the fact that all included patients were suffering from GERD symptoms. Preoperative data on esophageal functional testing were not provided in this study [23].

Two smaller case series compared pre- and postoperative functional esophageal testing in OAGB patients with a short-term follow-up of twelve months. The first study by Tolone S. et al. with 15 OAGB patients also found a decrease in acid reflux and normal esophageal motility but, different to our study, low rates of biliary reflux. This difference may have been caused by the shorter follow-up, as after twelve months, the intraabdominal pressure is at the lowest point after surgery [13]. On the other hand, the second study of eleven patients by Doulami G. et al. found a decrease in acid reflux but also a slight increase in non-acid reflux episodes, similar to our study [24].

The anti-reflux sutures that were performed in all patients in this study were described first by Carbajo M. [25] as mechanism to prevent biliary reflux. A current observational study by Slagter N. et al. of 703 patients showed that applying these sutures leads to much lower conversion rates to RYGB [26]. Nevertheless, further objective studies dealing with this issue have to be awaited.

Comparing the results of this study to the literature available on SG [27], one can summarize that acid reflux is a problem in the long-term follow-up after SG but seems to improve after OAGB. Nevertheless, the increasing rates of non-acid reflux after OAGB are a potentially dangerous finding that must be kept in mind. Genco A. et al. compared GERD symptoms and endoscopic findings after SG, RYGB, and OAGB in a long-term follow-up. They found that RYGB was superior to OAGB and SG, not only in terms of GERD symptoms but also when monitoring Gastritis, Barrett´s esophagus, anastomotic inflammation, and biliary reflux to the stomach and esophagus. Only in terms of marginal ulcers, OAGB and RYGB were not significantly different [28]. On the other hand, the latest update (10 years of follow-up) of the SLEEVEPASS trial by Salminen et al. showed that both in SG and RYGB Barrett´s esophagus rates were low (4%), even with much higher symptomatic de-novo reflux rates. Interestingly, the Barrett’s esophagus rate after SG was lower than in previously published long-term data studies [29].

Further, in a study comparing patients who had different bariatric procedures by the use of scintigraphy, Eldredge T.A. et al. showed biliary reflux to the pouch in 5% of the patients after RYGB, 70% after OAGB, and 31% after SG. The follow-up in this study was 6 months [30]. Based on the literature, it seems that RYGB is the best procedure in terms of preventing reflux. However, studies with larger patient cohorts have yet to be published.

As stated in a review by Zikos T.A. et al., non-acid symptoms cannot be differentiated clinically from other esophageal symptoms; therefore, impedance-24 h-pH-metry is needed for the diagnosis. Further, there is also no clear consensus regarding the definition of primary non-acid reflux [31]. Li D. et al. addressed the potential risk of biliary reflux by studying the association between gastric cancer and bile reflux in 30,465 patients who had gastroscopy. They found that bile reflux was closely associated with the development of gastric cancer and precancerous lesions. I.e., a higher rate of bile reflux may be related to a higher risk of developing gastric cancer [32]. A basic science study by Hong J. et al. assessing human Barrett’s cell lines found that bile acid damages the mucosa and induces long-term oxidative stress and cellular DNA damage, which can lead to esophageal adenocarcinoma [33].

Data on objective functional esophageal testing available in the literature today are based on a few OAGB patients only. It has been shown that one has to differentiate between revisional and non-revisional OAGB patients and also between short- and long-term follow-up when evaluating these data. Further studies should focus on comparing esophageal functional testing before and after different types of bariatric surgery (e.g., OAGB, RYGB, SG) with a similar follow-up.

### Outcome and quality of life

Compared to other bariatric procedures, e.g., Sleeve Gastrectomy, the results of SF-36 in all categories, as well as of BQL and GIQLI, are much higher after OAGB [34]. The same goes for the more objective outcome of the BAROS [35]. Comparing primary OAGB patients in this study to revisional OAGB patients in the literature, all scores (SF-36, BQL, GIQOL, and BAROS) were much higher in this study [36].

A study by Katayama R.C. et al. using SF-36 before and 6 months after OAGB showed a significant increase in QOL in nearly all categories, which seems to remain stable until the mid-term follow-up of 5.1 years in the present study [37]. Another study utilizing the GIQLI 36 months after OAGB found an even higher score (125 points) than the present study, maybe due to the slightly shorter follow-up [38]. Further, a study by Jain M. et al. reported results of the BAROS score (with comorbidities) 5 years after OAGB at a similarly high level of 6.84 points as in the present study [39]. To summarize, quality of life in the current study can be seen as relatively high, please note, however that no preoperative data were available.

### Limitations of the study

A limitation of this study is the small number of patients; however, we included all patients whose preoperative results of functional testing were available and that were willing to repeat these very uncomfortable postoperative examinations solely for the purposes of this study. This also leads to a possible/likely risk of positive or negative selection bias as patients with GERD symptoms may have been more willing to do esophageal functional testing again, or the other way around. Further, data on patients that did not agree to be included in this study cannot be provided in this publication, even if this might clarify the risk of bias.

Additionally, the two patients who had hiatal hernia repair intraoperatively might have influenced the outcome of this study.

A further limitation of this study is that the QOL questionnaires were not performed preoperatively, so a comparison of pre- to the postoperative ones was not possible. Nevertheless, these QOL data can be compared to the literature.

## Conclusion

This study has shown decreasing rates of acid reflux and an increase in non-acid reflux after a mid-term outcome in primary OAGB patients. Nevertheless, in the gastroscopy, signs of chronic irritation of the gastrojejunostomy, pouch, and distal esophagus were found, even in asymptomatic patients. Follow-up gastroscopies in OAGB patients after 5 years plus may be considered. Regarding weight loss, remission of AMP, and QOL, the results in this study were very good, indeed.
